# The Screening Research of NF-*κ*B Inhibitors from Moutan Cortex Based on Bioactivity-Integrated UPLC-Q/TOF-MS

**DOI:** 10.1155/2019/6150357

**Published:** 2019-03-03

**Authors:** Yujie Lu, Wenjuan Liu, Man Zhang, Yanfang Deng, Min Jiang, Gang Bai

**Affiliations:** State Key Laboratory of Medicinal Chemical Biology, College of Pharmacy and Tianjin Key Laboratory of Molecular Drug Research, Nankai University, Haihe Education Park, 38 Tongyan Road, Tianjin 300353, China

## Abstract

Inflammation is a common and important pathological process, and nuclear factor-*κ*B (NF-*κ*B) is a key mediator of it. Moutan Cortex (MC), the dried root cortex of* Paeonia suffruticosa* Andr., is widely used as a remedy for the treatment of inflammatory diseases in Asian region. However, there are few studies on the systematic identification of NF-*κ*B inhibitors of MC. In this study, the effect of inhibiting NF-*κ*B activation of MC was assessed at the cellular level using a tumor necrosis factor-*α* (TNF-*α*) induced inflammatory model. Subsequently, ultra-performance liquid chromatography-quadrupole/time of flight-mass spectrometry (UPLC-Q/TOF-MS) combined with biological activity assay was established to screen and identify potential anti-inflammatory ingredients in MC. The results revealed that MC significantly inhibited the activation of NF-*κ*B. Seven potential NF-*κ*B inhibitors were screened from MC, including oxypaeoniflorin, paeoniflorin, galloylpaeoniflorin, benzoyloxypaeoniflorin, mudanpioside C, gallic acid, and paeonol. Among them, the NF-*κ*B inhibitor activity of galloylpaeoniflorin, benzoyloxypaeoniflorin, and mudanpioside C is first reported here. In conclusion, the anti-inflammatory activity of MC was associated with the seven components mentioned above. And the bioactivity-integrated UPLC-Q/TOF which contains both chemical and bioactive details is suitable for screening active ingredients from natural medicines.

## 1. Introduction

Inflammation is a multicomponent response to injury, tissue stress, and infection, associated with most diseases, and it can occur in various tissues and organs of the organism [1]. Clinically, there are two main categories of anti-inflammatory drugs: steroidal drugs and non-steroidal anti-inflammatory drugs (NSAIDs). Steroidal drugs generally refer to adrenocortical hormones, which have strong anti-inflammatory effect, but have obvious side effects such as water-sodium retention, puffiness, and osteoporosis [2]. And NSAID is one of the most widely used drugs in the world and are mainly classified into salicylates, propionic acids, indoles, fenamic acids, acetic acids and pyrazolones [3]. However, it has potential cardiovascular and gastrointestinal bleeding risks [4]. Due to the strong toxic and side effects of many synthetic drugs, and because traditional Chinese medicine (TCM) has the advantages of abundant resources, definite therapeutic effectiveness, and fewer side effects, the discovery of novel anti-inflammatory drugs from natural compounds has gradually become a research hotspot [5].

Moutan Cortex (MC) is the dried root cortex of* Paeonia suffruticosa* Andrews and commonly used for removing blood stasis, dredging meridian, expelling pus, and eliminating inflammation in TCM prescriptions [6, 7]. Some pharmacological studies have showed that MC could inhibit the production of NO and tumor necrosis factor-*α* (TNF-*α*) induced by lipopolysaccharide/recombinant interferon-*γ* (LPS/rIFN-*γ*) [8]. In animal experiments, MC could significantly decrease the level of cytokines including interleukin-1 (IL-1), macrophage inflammatory peptide-2 (MIP-2), IL-6, and IL-10, which apparently inhibited LPS-induced acute lung injury in rat models [9]. Available evidence revealed that MC had the potential NF-*κ*B inhibitory activity and clinical anti-inflammatory efficacy; however, previous studies primarily focused on the single component, and the integral active components and their mechanisms of action have not yet been fully clear. Therefore, we mainly conducted a systematic study of the potential NF-*κ*B inhibitors in MC.

In the current study, dual-luciferase reporter assay integrated UPLC-Q/TOF-MS was utilized to screen out potential NF-*κ*B inhibitors of MC extract. And the anti-inflammatory activity of some components was confirmed through in vitro experiments. Additionally, the mechanism of active compounds was predicted through network pharmacology methods.

## 2. Materials and Methods

### 2.1. Chemicals and Reagents

Gallic acid, paeonol, and paeoniflorin were purchased from Macklin Biochemical Co., Ltd. (Shanghai, China). Dexamethasone (Dex) was obtained from Sigma Chemical Co. (St Louis, MO, USA). HPLC-grade formic acid was purchased from Meryer Chemical Technology Co., Ltd. (Shanghai, China). All the above chemical reagents were HPLC≥98%. Acetonitrile hypergrade for UPLC-Q/TOF-MS was acquired from Merck KGaA (Darmstadt, Germany). The polyethylenimine (PEI) transient transfection reagent was purchased from PolyScience (Carlsbad, CA, USA). Dual-Luciferase® Reporter Assay System 10-Pack was obtained from Promega (Madison, WI, USA). Both interleukin-6 (IL-6) and interleukin-1 beta (IL-1*β*) Human ELISA Kit were purchase from Abcam (Cambridge, UK). All reagents for cell culture were obtained from biological industries (Israel).

### 2.2. Drug Materials and Sample Preparation

MC was acquired from the Anguo Chinese herbal medicine market and identified by Professor Zhang Tiejun from Tianjin Pharmaceutical Research Institute. The dried MC was crushed into powder, weighing the powder accurately. Then, add methanol (W/V=1:20), ultrasonic extraction of 30 min at 25°C, centrifuge and collect the supernatant. The extract was dried into powder by vacuum freeze-drying.

### 2.3. Cell Culture

The HEK 293 cells were purchased from American Type Culture Collection (Rockville, MD, USA) and were cultured in DMEM high glucose with 10% FBS, 100 IU/mL penicillin, and 100 mg/mL streptomycin at 37°C and 5% CO_2_ in a thermostatic incubator. BEAS-2B, derived from human bronchial epithelial cells, was also purchased from the American Type Culture Collection (Rockville, MD, USA). Their culture conditions were the same as those of HEK 293 cells, except for DMEM/F-12 (HAM) 1:1 instead of DMEM high glucose.

### 2.4. Verify the Anti-Inflammatory Activity of MC

HEK 293 cells were cotransfected with the NF-*κ*B luciferase reporter plasmid pGL 4.32 (Promega WI, USA) and Renilla plasmid for 22 h when cell density reached 60 %, and PEI was used as the transfection reagent according to the manufacturer's instructions. And the cells were randomly assigned to six groups (n=4): control group, model group, Dex (5×10^−5^ mol/L) group, MC-H (1 mg/mL) group, MC-M (0.1 mg/mL) group, and MC-L (0.01 mg/mL) group. All the cells except for cells in the control group were stimulated with tumor necrosis factor-*α* (TNF- *α*, 20 ng/mL) and simultaneously given with drugs for 6 h. Among them, Dex was used as a positive control and could decrease the expression of NF-*κ*B in HEK 293 cells. Relative content of NF-*κ*B in each group was represented as relative light unit (RLU) ratio following the manufacturer's instructions.

### 2.5. UPLC-Q/TOF-MS Analysis

A Waters Acquity UPLC System (Waters Co., Milford, MA, USA) equipped with a photo diode array detector (PDAD) was used to analyze samples. The chromatographic column was an Acquity BEH C_18_-column (2.1 mm ×100 mm, 1.7 *μ*m; Waters Co.). The flow rate was 0.40 mL/min and the column temperature was maintained at 30°C. The test sample injection volume was 5 *μ*L. The optimal mobile phase was composed of A (0.1% formic acid in water) and B (acetonitrile) and the ratio was as follows: 0-1.0 min, 2% to 5% B; 1.0-2.0 min, 5% to 6% B; 2.0-4.0 min, 6% to 7% B; 4.0-6.0 min, 7% to 9% B; 6.0-9.0 min, 9% to 14% B; 9.0-12.0 min, 14% to 16% B; 12.0-18.0 min, 16% to 21% B; 18.0-21.0 min, 21% to 27% B; 21.0-22.0 min, 27% to 40% B.

Accurate mass and MS/MS measurements were performed by a Waters Q/TOF micro Synapt High Definition Mass Spectrometer (Waters MS Technologies, Manchester, UK) with a dual electrospray ionization (ESI) system. The optimal analytical condition was set as follows: the source temperature was 110°C; the capillary voltage was 3.0 kV in positive ion mode and 2.5 kV in negative ion mode; the sample and extraction cone voltage were 30 V and 4.0 V, respectively; the flow rate of the desolvation gas was 600 L/h at a desolvation temperature of 350°C; the cone gas flow was 50 L/h. The MS spectra scanning range in the wide-pass mode was 50 Da to 1200 Da. Leucine enkephalin amide acetate (LEA, 200 ng/mL) was used as the lock mass ([M+H]^+^ = 555.2931, [M-H]^+^ = 553.2775). And a flow rate was set at 20 *μ*l/min. Based on the MS/MS information, peaks of interest were confirmed by the molecular weight and structure of the contained constituent. Some peaks with the similar MS/MS information could be identified by their different retention behaviors.

### 2.6. Sample Preparation for Activity Assay

The freeze-dried powder of MC extract was accurately weighed and ultrasonic dissolved in methanol (1 mg/mL). After column separation, the 10% fractions were transported to the Q-TOF/MS system for components identification and the 90% fractions were collected every 0.5 min into a deep 96-well plate and vacuum dried at 56°C. The residues were dissolved in DMEM (50 *μ*L) for dual-luciferase assay. The operation process was the same as above. Screening out fractions that could reduce the level of NF-*κ*B was performed for further structural analysis.

### 2.7. Verification of Monomer Compounds Activity

Dual-luciferase assay system was used to verify the anti-inflammatory activity of the monomeric compounds. The operation steps were the same as above. Additionally, human IL-6 and IL-1*β* ELISA kits were used, respectively, to measure the concentrations of inflammatory factors (IL-6 and IL-1*β*) in the culture supernatants of BEAS-2B cells after the stimulation of drugs and TNF-*α*. The absorbance of each sample was measured at 450 nm using a Bio-Rad Model 680 microplate reader. Data processing followed the instructions of ELISA kits.

### 2.8. Statistical Analysis

The test results were represented with mean ± SEM. And t-test was used for comparison of significant differences among different groups. SPSS v.18.0 statistical analysis software (SPSS Inc., Chicago USA) was used for statistical analysis. Results with values of* P* < 0.05 were considered statistically significant.

## 3. Results

### 3.1. Effects of MC on NF-*κ*B Inhibition

**Figure 1:**
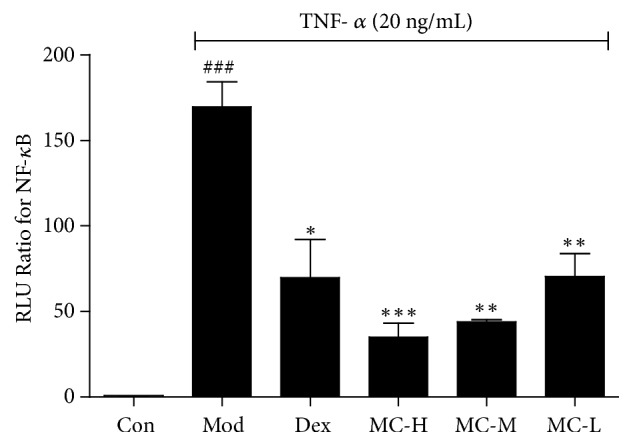
Effects of the three doses of MC on the level of NF-*κ*B in TNF-*α* induced HEK 293 cells. Values are presented as the mean ± SEM; n=5 per group. ###* P*< 0.001 compared to the control group; *∗ P*< 0.05, *∗∗ P*< 0.01, *∗∗∗ P*< 0.001 compared to the model group.

To verify the effects of MC on anti-inflammatory, the level of NF-*κ*B in TNF-*α* induced HEK 293 cells was investigated using a dual-luciferase reporter assay system. As shown in Figure 1, the different doses of MC (MC-L, 0.01 mg/mL; MC-M, 0.1 mg/mL; MC-H, 1 mg/mL) not only significantly inhibited NF-*κ*B production, but also showed a dose-dependent inhibition (*P*<0.05). The result confirmed that MC contained some components with potential anti-inflammatory activity.

### 3.2. Bioactivity Screening and Components Identification of MC

**Figure 2:**
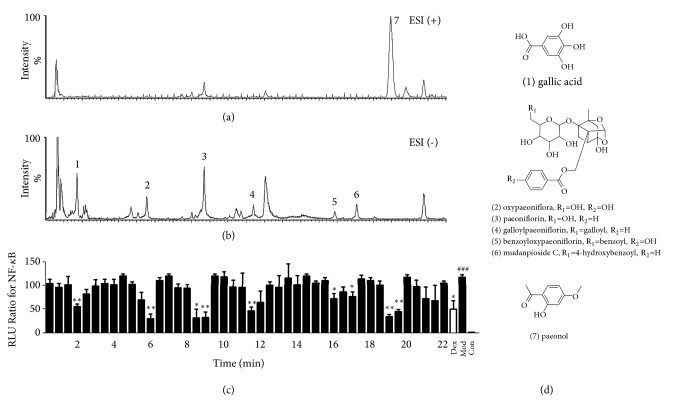
UPLC-Q/TOF-MS and bioactivity analysis of MC. (a and b) Base peak intensity (BPI) chromatograms of MC in the ESI positive and negative, respectively. (c) Bioactivity chromatograms obtained via the dual-luciferase reporter assay for NF-*κ*B inhibition activation. (d) Chemical structures of the bioactive compounds in MC. Values are presented as the mean ± SEM; n=5 per group. ###* P*< 0.001 compared to the control group; *∗ P*< 0.05, *∗∗ P*< 0.01, *∗∗∗ P*< 0.001 compared to the model group.

To identify the anti-inflammatory components in MC, we performed a dual-luciferase reporter assay integrated UPLC-Q/TOF-MS. Representative chemical components of MC and the total ion current chromatograms in positive and negative ESI modes, respectively, are shown in Figures 2(a) and 2(b). In total, seven fractions showed potential NF-*κ*B inhibitor activity (Figure 2(c)). We analyzed the seven fractions, and compounds they contain were identified by exact molecular weights and diagnostic fragment ions (Figure 2(d)). The detailed results and MS/MS information were shown in Table 1. The seven potential NF-*κ*B inhibitors could be classified into two types according to their chemical structures: monoterpenes (oxypaeoniflorin, paeoniflorin, galloylpaeoniflorin, benzoyloxypaeoniflorin, mudanpioside C) and phenolic acids (gallic acid, paeonol). Among them, the NF-*κ*B inhibitory activities of galloylpaeoniflorin, benzoyloxypaeoniflorin, and mudanpioside C are first reported here. We then tested and verified the anti-inflammatory activity of these monomers.

### 3.3. Verification of NF-*κ*B Inhibitor Activity of Monomeric Compounds

**Figure 3:**
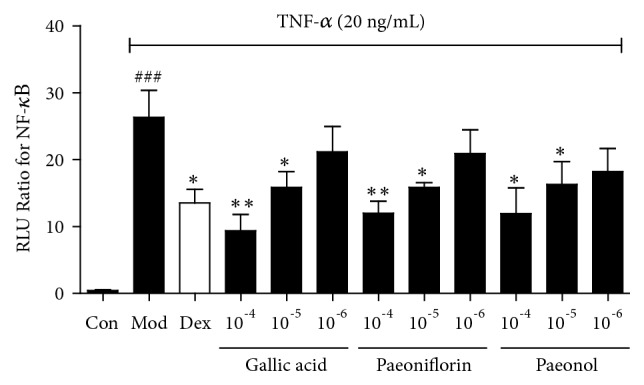
Confirmation of bioactive compounds from MC by the dual-luciferase reporter assay system. Values are presented as the mean ± SEM; n=5 per group. ###* P*< 0.001 compared to the control group; *∗ P*< 0.05, *∗∗ P*< 0.01, *∗∗∗ P*< 0.001 compared to the model group.

Oxypaeoniflorin, galloylpaeoniflorin, benzoyloxypaeoniflorin, and mudanpioside C have the same structural characteristics, all of which have paeoniflorin as the mother nucleus structure. Since the generation of drug efficacy mainly depends on their chemical structure, we selected paeoniflorin, the mother nucleus of the four compounds, as a representative component to study their anti-inflammatory activity. For verification of NF-*κ*B inhibitory activity, varying concentrations (10^−4^ mol/L, 10^−5^ mol/L, and 10^−6^ mol/L) of three ingredients (gallic acid, paeoniflorin, and paeonol) were chosen for dual-luciferase reporter assay. As shown in Figure 3, gallic acid, paeoniflorin, and paeonol all showed significant NF-*κ*B inhibitory effects.

### 3.4. Confirmation of the Bioactivity of NF-*κ*B Inhibitors

**Figure 4:**
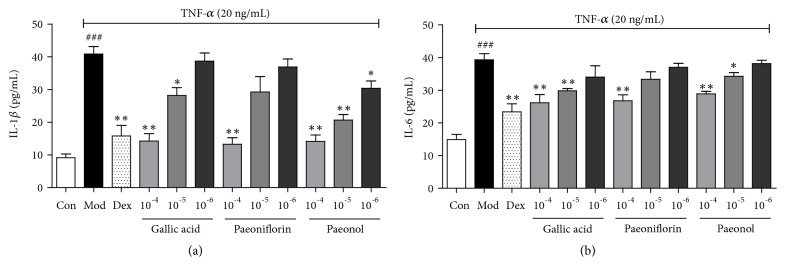
Confirmation of the effects by potential NF-kB inhibitors. (a and b) IL-1*β* and IL-6 expression in TNF-*α* induced BEAS-2B cells, respectively. Values are presented as the mean ± SEM; n=5 per group. ###* P*< 0.001 compared to the control group; *∗ P*< 0.05, *∗∗ P*< 0.01, *∗∗∗ P*< 0.001 compared to the model group.

For further verification of anti-inflammatory activity, gallic acid, paeoniflorin, and paeonol with the same concentration gradient (10^−4^ mol/L, 10^−5^ mol/L, and 10^−6^ mol/L) were selected to test their effects on the expression of IL-6 and IL-1*β* in TNF-*α* induced BEAS-2B cells. As shown in Figure 4, all these three compounds could inhibit the overexpression of inflammatory factors in a dose-dependent manner. Additionally, the results also illustrated the correctness of the screening method.

## 4. Discussion

The transcription factor NF-*κ*B acts a key role in the process of immune response and it can usually be activated when exposed to inflammatory cytokines such as TNF-*α*, viral infection, ultraviolet irradiation, and other physiological and nonphysiological stimuli [10–12]. However, the activated NF-*κ*B signaling pathway participates not only in immune regulation and inflammation, but also in infection, cell cycle regulation, cell differentiation, and apoptosis [13–15]. If the activation cannot be eliminated in time, it may lead to serious pathological reactions, such as rheumatoid arthritis, systemic lupus erythematosus, septic shock, atherosclerosis, and cancer [16–18]. Consequently, inhibitors of NF-*κ*B activation are of primary significance in protecting cells from the potential damage of inflammation.

**Figure 5:**
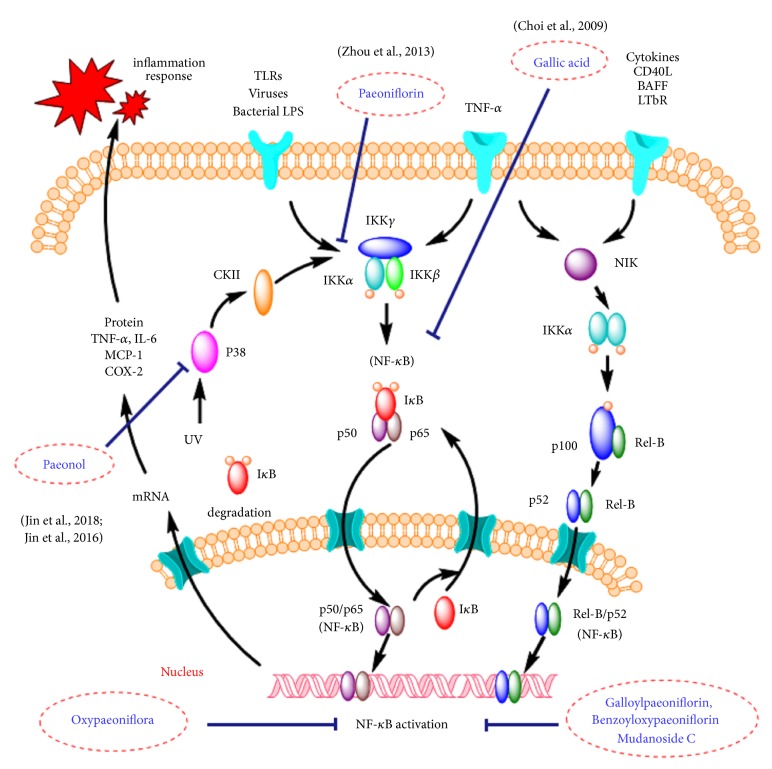
Molecular mechanism of MC on anti-inflammatory effect.

Based on the efficient separation and analysis functions of UPLC-Q-TOF-MS and cell biological method, seven compounds with NF-*κ*B inhibitory activity were screened from MC. Among them, gallic acid could inhibit NF-*κ*B activation by prevention of RelA acetylation [19]. And paeoniflorin could significantly inhibit NF-*κ*B by reducing the expression of the phosphorylation of I*κ*B*α* and p65 [20]. Additionally, paeonol could suppress NF-*κ*B signaling through blocking MAPK/p38 signaling pathway [21, 22]. Furthermore, oxypaeoniflorin could inhibit the elevation of the expression levels of NF-*κ*B although the mechanism remained unclear [23]. Additionally, NF-*κ*B inhibitory activity of galloylpaeoniflorin, benzoyloxypaeoniflorin, and mudanoside C has not been reported in previous studies. In the present study, those three compounds could inhibit the activation of NF-*κ*B induced by TNF-a and could be considered as novel NF-*κ*B inhibitors. In general, the efficacy of drugs depends mainly on their chemical structures [24]. According to our results, other monoterpenoids with paeoniflorin as the core structure may also have NF-*κ*B inhibitory activity, which can be used as a lead compound for the study of innovative drugs. Furthermore, the results demonstrated that the anti-inflammatory activity of MC was related to the process of various components acting on multiple targets (Figure 5), which was consistent with the characteristics of TCMs with multiple components, multiple pathways, and multiple targets [25, 26].

In addition to its anti-inflammatory effects, MC also has cytotoxicity to cancer cells. Recent research has showed that MC extract could reduce cell viability with IC50 within 1~2 mg/ml in bladder cancer cells [7]. And after treatment for 48 h with paeonol (400 *μ*g/ml), one of the active ingredients of MC, the ratio of apoptotic cells reached 34.79% [27]. This study demonstrated that, at a concentration of 0.01 mg/mL, the extract of MC already had anti-inflammatory activity. Taking paeonol as an example, it showed significant NF-*κ*B inhibitory effects at a concentration of 10^−5^ mol/L, which was much lower than its toxic content. This finding was in agreement with the characterization of most drugs as playing a therapeutic role in a certain dose range.

The quality marker (Q-marker) representing the quality of TCM should not only take the content of certain components as an index, but also be able to reflect its efficacy [28, 29]. Consequently, determining the content of paeonol as the only approach to evaluate the quality of MC in Chinese Pharmacopeia is unilateral. According to our results, oxypaeoniflorin, paeoniflorin, galloylpaeoniflorin, benzoyloxypaeoniflorin, mudanpioside C, gallic acid, and paeonol were related to the anti-inflammatory effect of MC and could be considered as a reference standard for evaluating the quality of MC.

## 5. Conclusions

In conclusion, MC showed significant efficacy in inhibiting NF-*κ*B activation, and seven bioactive components were screened by a dual-luciferase reporter assay integrated UPLC-Q/TOF-MS. According to their structural characteristics, the potential NF-*κ*B inhibitors could be categorized into two types: monoterpenes (oxypaeoniflorin, paeoniflorin, galloylpaeoniflorin, benzoyloxypaeoniflorin, mudanpioside C) and phenolic acids (gallic acid, paeonol). Thereinto, galloylpaeoniflorin, benzoyloxypaeoniflorin, and mudanpioside C were first reported to the effects of inhibiting the activation of NF-*κ*B. The present study demonstrates that MC contains a variety of structurally diverse anti-inflammatory active ingredients, acting on different targets, which provides a basis for the discovery of novel anti-inflammatory drugs with fewer side effects. And this article may provide a useful reference for improving the quality standards of MC in the future. Additionally, these experimental results also showed that the bioactivity-integrated UPLC-Q/TOF which contain both chemical and bioactive details is suitable for screening active ingredients from natural medicines.

## Figures and Tables

**Table 1:** MS/MS data in (±) ESI modes and the identification results for the bioactive compounds in Moutan Cortex (MC).

No.	RT/min	Identification results	Composition	m/z	MS/MS
1	1.71	gallic acid	C_7_H_6_O_5_	169.0145 [M-H]^−^	125 [M-H-COO]^−^
2	5.55	oxypaeoniflora	C_23_H_28_O_12_	495.1476 [M-H]^−^	465 [M-H-CH_2_O]^−^
					137 [M-H-C_6_H_11_O_6_-C_10_H_12_O_3_]^−^
3	8.69	paeoniflorin	C_23_H_28_O_11_	479.1545 [M-H]^−^	525 [M+HCOO]-
					449 [M-H-HCHO]^−^
					327 [M-H-HCHO-C_7_H_5_O_2_]^−^
					121 [M-H-C_10_H_11_O_3_-C_6_H_11_O_6_]^−^
4	11.41	galloylpaeoniflorin	C_30_H_32_O_15_	631.1708 [M-H]^−^	469 [M-H-C_9_H_6_O_3_]^−^
5	15.88	benzoyloxypaeoniflorin	C_30_H_32_O_13_	599.1768 [M-H]-	569 [M-H-CH_2_O]^−^
					431 [M-H-C_7_H_5_O_3_]^−^
6	17.09	mudanpioside C	C_30_H_32_O_13_	599.1768 [M-H]^−^	569 [M-H-CH_2_O]^−^
					477 [M-H-C_7_H_5_O_2_]^−^
7	18.98	paeonol	C_9_H_10_O_3_	167.0711 [M+H]^+^	149 [M+H-H_2_O]^+^

## Data Availability

The data used to support the findings of this study are included within the article.
